# Golden Rules of Diseases of Children

**Published:** 1902-09

**Authors:** 


**Golden Rules of Diseases of Children.** By George Carpenter, M.D. Pp. 101. Bristol: John Wright & Co. [1901.]

This book shows a thorough knowledge of diseases of children both from the clinical and pathological point of view, as well as an intelligent grasp of important facts. One cannot glance at any page without having some point of value impressed upon the mind. In these days of superabundance of medical literature such books fill a useful place. They bring to the surface what the practical wish to know.

